# Awareness of Diabetes and Risk Factors for Diabetic Retinopathy Among Patients Attending the Eye Outpatient Department of a Tertiary Care Centre in Northeast India

**DOI:** 10.7759/cureus.98382

**Published:** 2025-12-03

**Authors:** Nabajani Dutta, Bharati Basumatari, Anjan J Bhuyan, Krishangi Kashyap, Shubra Das, Harsha Bhattacharya, Putul Mahanta

**Affiliations:** 1 Nursing, Royal School of Nursing, Guwahati, IND; 2 Radiology, Nalbari Medical College and Hospital, Nalbari, IND; 3 Otolaryngology - Head and Neck Surgery, PA Sangma International Medical College, Khanapara, IND; 4 Oncology Nursing, Nalbari Medical College and Hospital, Nalbari, IND; 5 Ophthalmology, Gauhati Medical College, Guwahati, IND; 6 Ophthalmology, Sri Sankaradeva Nethralaya, Guwahati, IND; 7 Forensic Medicine and Toxicology, Nalbari Medical College, Nalbari, IND

**Keywords:** diabetes mellitus, diabetic retinopathy, diabetics, lifestyle factors, non-diabetics

## Abstract

Introduction

Over half a billion individuals worldwide suffer from diabetes mellitus (DM). Among people of working age, diabetic retinopathy (DR) is the leading cause of blindness and one of the most prevalent side effects of DM. DR screening and care in this part of the country are severely hampered by a lack of awareness of the consequences of diabetes and by infrequent screening, especially among individuals with diagnosed diabetes. The present study aims to assess the awareness of DM and its risk factors for DR and to identify the associated factors among patients attending the eye outpatient department (OPD) of a tertiary care centre in northeast India.

Method

The present hospital-based cross-sectional study included 1757 patients aged 23 years or older with or without DM and DR who visited the eye OPD at a tertiary care centre in Northeast India. A structured questionnaire was used to collect the data. The collected data were analysed by age group and diabetes mellitus status. A z-test for two proportions and a chi-square test were used to determine whether there was a significant difference or association between variables. A p-value <0.05 was considered significant, while a p-value <0.01 was considered highly significant.

Results

The participants' ages ranged from 23 to 94 years, with a male:female ratio of 1.35:1, among whom 44.2% (n=777) were diabetic. The most frequently stated risk factors among people with diabetes were physical inactivity (83.7%, n=650), family history of diabetes (77.5%, n=602) and being overweight (71.4%, n=555). A considerably greater percentage of people with diabetes (n=724, 93.2%) recognised blurred vision as a symptom of DM than non-diabetics. The younger age group (those under 55) was more knowledgeable about most DM risk factors and symptoms. Over 90% of people with diabetes (n=710) were aware of blood tests for diabetes monitoring, but only 45% (n=350) knew the normal range of postprandial blood sugar (PPBS) levels. Both diabetics and non-diabetics have relatively low awareness of the typical ranges of fasting blood sugar (FBS) and HbA1c. There was no discernible variation in the age groups' awareness of the different aspects of diabetes monitoring. There was a significant correlation found between the patients' age, DM status, religion, place of residence, family type, marital status, and socio-economic status with awareness of DM and DR.

Conclusion

Most participants had moderate to high knowledge of DM and its risk factors for DR. People who had previously had DM were significantly more knowledgeable about the disease's signs and risk factors. However, both diabetics and non-diabetics were less aware of the lifestyle risk factors for DM. Various socio-demographic factors influence patients' awareness of DM and DR. The participants' awareness levels vary by age and diabetes mellitus status.

## Introduction

Metabolic syndrome is an increasingly common condition that predisposes individuals to diabetes mellitus (DM) [1]. DM affects around half a billion people globally and a projected 783 million people will have diabetes by 2045 [2]. Over 90% of people with diabetes have type 2 DM, which is impacted by socio-economic, demographic, environmental, and genetic factors. Rapid urbanisation, an ageing population, a sedentary lifestyle, and a rise in the prevalence of overweight and obesity all contribute to the rise of type 2 DM [3]. As of 2019, India has 77 million adults with diabetes, making it the second highest in the world. The number of those afflicted has been rising rapidly, representing a significant public health burden [4].

Diabetic retinopathy (DR) is one of the most common consequences of DM and the primary cause of blindness in the working-age population, which typically shows no symptoms until it is too late [5,6]. People with diabetes in India had a 16.9% prevalence of DR and a 3.6% prevalence of sight-threatening DR (STDR) [7]. Every person with diabetes should ideally undergo routine vision-threatening DR screening. Early screening and awareness need to be provided by educated medical professionals and retina specialists. However, inequitable care delivery and low disease knowledge are two of India's biggest challenges [8]. The majority of patients who present to India's eye care institutions with advanced and irreversible diabetic eye disease are due to low utilisation of healthcare facilities caused by a lack of knowledge about available treatment alternatives and inadequate physician referrals. Awareness of DM and DR can be raised through practice-orientated education, promoting facilities tailored to the appropriate demographics at each level of an eye health pyramid, and continuing fundus and glucose screening programmes [9]. Lack of knowledge of the need for regular DR screening has turned many patients into the advanced stage of DR before their first eye check-ups. A study in Nepal found that although patients with diabetes are generally aware of the negative visual effects of DR, their readiness to undergo retinal examinations was inadequate [10]. Often, patients view DR screening as basic eye exams that include vision tests, refraction measurements, and non-dilated pupil exams [11].

Despite being traditionally considered low risk due to active lifestyles and diet, diabetes prevalence in the northeastern states of India is steadily increasing due to urbanisation, changing diets, and reduced physical activity [12]. DR remains underdiagnosed in Northeast India due to poor access to ophthalmic care. Limited access to retinal care specialists in hilly and tribal areas, a lack of knowledge about the effects of diabetes, and infrequent screening even among those with confirmed diabetes are major challenges in DR screening and management in this part of the country.

The primary objective of the present study was to assess the level of awareness regarding DM and its risk factors for DR among patients attending the eye outpatient department (OPD) of a tertiary care centre in Northeast India. The secondary objectives were to identify socio-demographic factors associated with participants' levels of awareness regarding age and diabetes status. Thus, the present study aims to assess the level of awareness regarding diabetes and its risk factors for diabetic retinopathy and to identify associated factors among patients attending the eye OPD of a tertiary care centre in Northeast India. The diabetic group showed significantly greater awareness of the effects of diabetes on other organs. Regarding age group, participants aged 55 years and older showed significantly higher knowledge of various preventive measures for diabetes.

## Materials and methods

The present hospital-based cross-sectional study included 1757 patients who visited the Eye OPD of Sri Sankaradeva Netralaya (SSDN), Guwahati, Assam, between April 17, 2023, and June 29, 2024. Every patient was questioned about their awareness of diabetes and diabetic retinopathy, irrespective of their DM status. Written informed consent was obtained from all the participants. Ethical approval for this study was obtained from the Institutional Ethics Committee of SSDN, Guwahati, Assam (No. SSN/IEC/JANUARY/2019/21, dated January 12, 2019).

Inclusion and exclusion criteria

The study included participants aged 23-94 years with or without DM and DR who were willing to participate and provided consent. Severely ill or mentally incapacitated patients and patients with uncorrectable severe visual impairment or hearing loss were excluded.

Data collection tool

A structured questionnaire was used to collect the data (Annexure 1). The first part of the questionnaire collected demographic information for each participant, including age, gender, religion, family type, place of residence, marital status, history of diabetes mellitus, and dietary habits. Socio-economic status was determined using the updated, modified Kuppuswamy scale (2022), which is based on the head of the family's education and occupation level and the family's monthly income in rupees [13].

The second part of the questionnaire was designed to obtain information on participants' awareness of diabetes and risk factors for DR. It comprised 18 questions assessing participants' awareness of the characteristics, symptoms, diagnosis, management, treatment, and consequences of diabetes. The responses to the questions included both multiple-choice and single-choice options. Each correct response was scored as 1, while incorrect responses were scored as 0. The overall total awareness score was 48.

The questionnaire was initially developed in English and further translated into Assamese. Content validity was conducted by experts from various fields of medical sciences, and the final tool was implemented for data collection. The data collection tool was pre-tested for accuracy, completeness, and consistency on 5% of patients attending the eye OPD before data collection. Data collection commenced following changes to the tool based on pre-test results and content validity. The questionnaire was interviewer-administered by a single investigator. Data with incomplete participant information were excluded to avoid errors. The patients were given a pamphlet on diabetes and DR after completing the questionnaire.

Statistical analysis

The data analysis was conducted using Statistical Package for the Social Sciences (SPSS) version 22 (IBM Corp., Armonk, New York). The distributions of the variables were summarised using descriptive statistics, viz., frequency, percentage, mean, and standard deviation. The total awareness score for each participant was computed by adding all the individual response scores. The response proportions were compared using a z-test for two proportions for different variable categories. The level of awareness was further categorised into three categories - low, moderate, and high - based on the 50th and 75th percentiles of the data. The chi-square test was used to assess the potential association between awareness level and socio-demographic factors. A p-value <0.05 was considered significant, while a p-value <0.01 was considered highly significant.

The data were analysed separately by age group and by participants' diabetes mellitus status. The findings were combined and presented in a single table for comparison.

## Results

The study included 1757 patients who regularly visited the Eye OPD at SSDN in Guwahati, Assam. The participants' ages ranged from 23 to 94 years, with the majority (903, 51.4%) aged 54 years or older. The male:female ratio was observed to be 1.35:1. The proportion of known diabetics was 44.2% (n=777). Most of the participants (70.9%) were married (n=1246). Participants mostly followed Hinduism (n=1162, 66.1%), were from urban areas (n=1111, 63.2%), and belonged to nuclear families (n=1164, 66.2%). More than half (51.1%) of the participants had a lower-middle-class (n=898) socio-economic status. Almost 85% (n=1490) of the participants were non-vegetarian (Table 1). 

**Table 1:** Socio-demographic profile of participants The data have been presented as frequencies (n) and percentages (%); total sample size, N=1757. SES: Socio-economic status

Variables	Categories	Total (%)
Age group	<55 years	854 (48.6%)
	≥55 years	903 (51.4%)
History of diabetes mellitus	No	980 (55.8%)
	Yes	777 (44.2%)
Gender	Female	748 (42.6%)
	Male	1009 (57.4%)
Religion	Hindu	1162 (66.1%)
	Christian	180 (10.2%)
	Others	154 (8.8%)
Family type	Joint	593 (33.8%)
	Nuclear	1164 (66.2%)
Place of residence	Rural	646 (36.8%)
	Urban	1111 (63.2%)
Marital status	Married	1246 (70.9%)
	Unmarried	289 (16.4%)
	Widow/ Divorced/ Separated	222 (12.6%)
Socioeconomic status	Upper	53 (3.0)
	Upper middle	406 (23.1%)
	Lower middle	898 (51.1%)
	Upper lower	367 (20.9%)
	Lower	33 (1.9%)
Dietary habit	Non-vegetarian	1490 (84.8%)
	Vegetarian	267 (15.2%)

Respondents' awareness regarding diabetes and risk factors for diabetic retinopathy as per age and diabetes mellitus status

Regarding the risk factors of diabetes, known diabetics showed significantly more awareness regarding various risk factors and symptoms of diabetes mellitus. Physical inactivity (83.7%, n=650), family history of diabetes (77.5%, n=602), and being overweight (71.4%, n=555) were the most common reported risk factors among people with diabetes. While both groups showed less awareness regarding behavioural risk factors, non-diabetics showed more awareness of the risk of alcohol consumption (z-test value 6.4, p<0.01). Compared to non-diabetics, a significantly higher proportion of people with diabetes identified blurred vision (n=724, 93.2%) as a symptom of diabetes mellitus. On the other hand, the age-group comparison revealed that the younger age group (below 55 years) was more aware of most risk factors and symptoms of diabetes mellitus. More than 75% of participants in both age groups were aware that diabetes mellitus can cause blurred vision (Table 2).

**Table 2:** Awareness regarding risk factors and symptoms of diabetes mellitus as per diabetes mellitus status and age of the participants (n=1757) The data have been represented as the frequency (n) of positive responses and as percentages (%); total sample N=1757. Response proportions were compared separately as per the age and diabetes status of the participants; DM: Diabetes mellitus; *p < 0.05 was considered statistically significant for z- test.

Variables	Categories	Diabetes status			Age		
		Non-diabetic (n=980)	Diabetic (n=777)	z test (p-value	<55 years (n=854)	>55 years (n=903)	z test (p-value
The risk factors for DM include	Family history	630 (64.3%)	602 (77.5%)	-5.9 (<0.01)	582 (68.1%)	650 (72.0%)	-1.7 (0.08)
	Being overweight	608 (62.0%)	555 (71.4%)	-4.1 (<0.01)	612 (71.7%)	551 (61.0%)	4.7 (<0.01)
	Physical inactivity	656 (66.9%)	650 (83.7%)	-7.9 (<0.01)	649 (76.0%)	657 (72.8%)	1.5 (0.12)
	Smoking	447 (45.6%)	331 (42.6%)	1.3 (0.20)	452 (45.9%)	326 (36.1%)	7.1 (<0.01)
	Alcohol	552 (56.3%)	318 (40.9%)	6.4 (<0.01)	506 (59.3%)	364 (40.3%)	7.9 (<0.01)
	Intake of Junk food	659 (67.2%)	496 (63.8%)	1.5 (0.13)	647 (75.8%)	508 (56.3%)	8.6 (<0.01)
	Women on oral contraceptive pills	278 (28.4%)	227 (29.2%)	-0.4 (0.69)	277 (32.4%)	228 (25.2%)	3.3 (<0.01)
Symptoms of high blood sugar include	Frequent urination	623 (63.6%)	647 (83.3%)	-9.2 (<0.01)	624 (73.1%)	646 (71.5%)	0.7 (0.47)
	Increased thirst	591 (60.3%)	632 (81.3%)	-9.5 (<0.01)	605 (70.8%)	618 (68.4%)	1.1 (0.27)
	Extreme hunger	566 (57.8%)	525 (67.6%)	-4.2 (<0.01)	530 (62.1%)	561 (62.1%)	-0.02 (0.97)
	Unexplained weight loss	463 (47.2%)	547 (70.4%)	-9.7 (<0.01)	521 (61.0%)	489 (54.2%)	2.9 (<0.01)
	Blurred vision	641 (65.4%)	724 (93.2%)	-13.9 (<0.01)	651 (76.2%)	714 (79.1%)	-1.4 (0.15)
	Dry mouth	510 (52.0%)	606 (78.0%)	-11.2 (<0.01)	483 (56.6%)	633 (70.1%)	-5.9 (<0.01)
	Recurrent urine infection	408 (41.6%)	530 (68.2%)	-11.1 (<0.01)	413 (48.4%)	525 (58.1%)	-4.1 (<0.01)

As shown in Table 3, more than 90% (n=710) of people with diabetes were aware of blood testing, but only 45% (n=350) were aware of the normal postprandial blood sugar (PPBS) range. Awareness of the normal ranges for fasting blood sugar (FBS) levels and glycated haemoglobin (HbA1c) is very low in both groups. No significant difference in awareness of the various components of diabetes monitoring was observed between age groups. In the prevention case, the diabetic group significantly perceived that dietary control (98.1%, n=762) and regular health check-ups (93.8%, n=729) can prevent diabetes. On the other hand, the non-diabetic group reported significantly more regular physical exercise (88.1%, n=863) and avoidance of alcohol and tobacco (59.1%, n=579) to prevent diabetes. Also, the diabetic group showed significantly greater awareness of the effects of diabetes on other organs. Additionally, participants aged 55 years and above showed higher awareness of various preventive measures for diabetes, whereas the younger age group was more aware that a prediabetic condition is reversible. Participants from both age groups were highly aware of the effect of diabetes on eye health (Table 3).

**Table 3:** Awareness on monitoring, prevention and the effect of diabetes mellitus on other organs as per diabetes mellitus status and age of the participants The data have been represented as the frequency (n) of positive responses and as percentages (%); total sample N=1757. Response proportions were compared separately by age and diabetes status; DM: Diabetes mellitus; FBS: Fasting Blood Sugar; PPBS: Postprandial blood sugar; *p < 0.05 was considered statistically significant for the test.

Awareness statement	Response (Yes)	Diabetes status			Age		
		Non-diabetic (n=980)	Diabetic (n=777)	z test (p-value)	<55 years (n=854)	>55 years (n=903)	z test (p-value
DM can be detected by	blood test	837 (85.4%)	710 (91.4%)	-3.8 (<0.01)	764 (89.5%)	783 (86.6%)	1.8 (0.07)
Normal range for FBS	70-110 mg/dl.	295 (30.1%)	265 (34.1%)	-1.7 (0.07)	264 (30.9%)	296 (32.8%)	-.8 (0.40)
Normal range for PPBS	140-200 mg/dl.	384 (39.2%)	350 (45.0%)	-2.5 (0.01)	340 (39.8%)	394 (43.6%)	-1.6 (0.10)
Normal range for % HbA1c level	>5.7 to < 6.5%	281 (28.7%)	191 (24.6%)	1.9 (0.05)	240 (28.1%)	232 (25.7%)	1.1 (0.25)
The risk of DM can be reduced by	Dietary control	910 (92.9%)	762 (98.1%)	-5.0 (<0.01)	793 (92.9%)	879 (97.3%)	-4.4 (<0.01)
	Regular physical exercise	863 (88.1%)	642 (82.6%)	3.2 (<0.01)	714 (83.6%)	791 (87.6%)	-2.4 (0.02)
	Avoiding intake of alcohol & tobacco	579 (59.1%)	415 (53.4%)	2.4 (0.02)	441 (51.6%)	553 (61.2%)	-4.0 (<0.01)
	Regular Health check-up	840 (85.7%)	729 (93.8%)	-5.5 (<0.01)	739 (86.5%)	830 (91.9%)	-3.6 (<0.01)
	Obese people are more likely to develop diabetes	587 (59.9%)	431 (55.5%)	1.9 (0.06)	542 (63.5%)	476 (52.7%)	4.6 (<0.01)
	Prediabetes is reversible	476 (48.6%)	412 (53.0%)	-1.8 (0.06)	537 (62.9%)	351 (38.9%)	10.1 (<0.01)
	Gestational diabetes occurs in pregnancy.	180 (18.4%)	177 (22.8%)	-2.3 (0.02)	201 (23.5%)	156 (17.3%)	3.2 (<0.01)
DM can cause long-term damage in the	Kidney	760 (77.6%)	659 (84.8%)	-3.8 (<0.01)	747 (87.5%)	672 (74.4%)	6.9 (<0.01)
	Eyes	863 (88.1%)	771 (99.2%)	-9.1 (<0.01)	794 (93.0%)	840 (93.0%)	-0.04 (0.96)
	Nerves	453 (46.2%)	491 (63.2%)	-7.1 (<0.01)	547 (64.1%)	397 (44.0%)	8.4 (<0.01)

There was no significant difference in perceived duration of the physician visit between diabetics and non-diabetics. Whereas the younger age group mostly perceived that a diabetes patient should visit a physician at least once a month (44.7%, n=382). A significant difference in awareness of diabetes treatment options was observed between diabetics and non-diabetics (chi-square value: 52.4, p<0.01) and between the two age groups (chi-square value: 24.7, p<0.01). Participants mostly reported that diabetes treatment includes a combination of insulin, oral drugs, and exercise (Table 4).

**Table 4:** Awareness treatment of diabetes mellitus as per the diabetes mellitus status and age of the participants The data has been represented as frequency (n) of positive response and percentage (%); total sample N=1757. Response proportions were compared separately by age and diabetes status; p < 0.05 was considered statistically significant for the chi-square test.

Awareness questions	Response categories	Diabetes status		Chi-square (p-value)	Age group		Chi-square (p-value)
		Non-diabetic (n=980)	Diabetic (n=777)		<55 years	>=55 years	
Diabetes patients should visit a physician at least	Once a month (n=664)	393 (40.1%)	271 (34.9%)	5.5 (0.14)	382 (44.7%)	282 (31.2%)	43.4 (<0.01)
	Once in 3 months (n=555)	294 (30.0%)	261 (33.6%)		264 (30.9%)	291 (32.2%)	
	Once a year (n=361)	199 (20.3%)	162 (20.8%)		144 (16.9%)	217 (24.0%)	
	Twice in a year (n=177)	94 (9.6%)	83 (10.7%)		64 (7.5%)	113 (12.5%)	
Treatment of diabetes includes	Insulin only (n=206)	163 (16.6%)	43 (5.5%)	52.4 (<0.01)	104 (12.2%)	102 (11.3%)	24.7 (<0.01)
	Oral Anti Diabetic Drugs (n=338)	171 (17.4%)	167 (21.5%)		124 (14.5%)	214 (23.7%)	
	Exercise (n=16)	9 (0.9%)	7 (0.9%)		10 (1.2%)	6 (0.7%)	
	All of the above (n=1197)	637 (65.0%)	560 (72.1%)		616 (72.1%)	581 (64.3%)	

Awareness scores for diabetes and its associated factors were calculated from responses to the awareness questionnaire. The mean overall awareness score (SD) was 29.8 (5.7). The total awareness scores were further divided into three categories based on the 50th and 75th percentiles. The 50th and 75th percentiles of the data were 29 and 34, respectively. Therefore, the categories are defined as low awareness (awareness score <29), moderate awareness (29<awareness score<34) and high awareness (awareness score ≥34). A high level of awareness of diabetes and its associated factors was observed in 526 patients (29.9%), while 551 patients (31.4%) had a moderate level of awareness (Table 5).

**Table 5:** Level of awareness on diabetes and risk factors for diabetic retinopathy among the participants (n=1757) The data has been represented as frequency (n) of positive response and percentage (%); total sample N=1757.

Awareness level	Frequency
Low awareness (<29)	680 (38.7%)
Moderate Awareness (29-33)	551 (31.4%)
High awareness (>33)	526 (29.9%)

Association of awareness of DM and awareness of diabetes, and risk factors for diabetic retinopathy with socio-demographic variables

The level of awareness was considerably low in the above-55 age group (44.2%, n=399/903) and among those with no history of DM (47.3%, n=464/980). A high level of awareness was noted among the Christian community (70.6%, n=127/180). Participants from a rural background (41.8%, n=270/646), those residing in nuclear families (39.5%, n=460/1164), and the widow/widower or separated group (57.2%, n=127/222) also showed lower awareness. The awareness level was considerably higher in the upper-middle-class group (40.6%, n=165/406), but 36 of 53 (67.9%) participants in the upper class showed a low level of awareness. The awareness levels were significantly associated with age group, history of diabetes mellitus, religion, place of residence, family type, marital status, and socio-economic status of the participants (Table 6).

**Table 6:** Association of awareness of diabetes mellitus with socio-demographic variables (n=1757). The data has been represented as frequency (n) and percentage (%); total sample N=1757. SES: Socio-economic status; p < 0.05 was considered statistically significant for the chi-square test

Variables	Categories	Awareness level categories			Chi-square value	p-value
		Low awareness (n=680)	Moderate awareness (n=551)	High awareness (n=526)		
Age-group	<55 years (n=854)	281 (32.9%)	278 (32.6%)	295 (34.5%)	26.9	<0.01
	>=55 years (n=903)	399 (44.2%)	273 (30.2%)	231 (25.6%)		
History of diabetes mellitus	No (n=980)	464 (47.3%)	255 (26.0%)	261 (26.6%)	71.0	<0.01
	Yes (n=777)	216 (27.8%)	296 (38.1%)	265 (34.1%)		
Gender	Female (n=748)	291 (38.9%)	250 (33.4%)	207 (27.7%)	4.0	0.13
	Male (n=1009)	389 (38.6%)	301 (29.8%)	319 (31.6%)		
Religion	Hindu (n=1162)	438 (37.7%)	431 (37.1%)	293 (25.2%)	189.0	<0.01
	Muslim (n=261)	113 (43.3%)	76 (29.1%)	72 (27.6%)		
	Christian (n=180)	46 (25.6%)	7 (3.9%)	127 (70.6%)		
	Others (n=154)	83 (53.9%)	37 (24.0%)	34 (22.1%)		
Place of residence	Rural (n=646)	270 (41.8%)	209 (32.4%)	167 (25.9%)	8.5	0.01
	Urban (n=1111)	410 (36.9%)	342 (30.8%)	359 (32.3%)		
Type of family	Joint (n=593)	220 (37.1%)	167 (28.2%)	206 (34.7%)	10.4	0.005
	Nuclear (n=1164)	460 (39.5%)	384 (33.0%)	320 (27.5%)		
Marital status	Unmarried (n=289)	92 (31.8%)	125 (43.3%)	72 (24.9%)	82.4	<0.01
	Married (n=1246)	461 (37.0%)	353 (28.3%)	432 (34.7%)		
	Widowed/widower/separated (n=222)	127 (57.2%)	73 (32.9%)	22 (9.9%)		
SES	Upper (n=53)	36 (67.9%)	15 (28.3%)	2 (3.8%)	65.4	<0.01
	Upper middle (n=406)	129 (31.8%)	112 (27.6%)	165 (40.6%)		
	Lower middle (n=898)	345 (38.4%)	292 (32.5%)	261 (29.1%)		
	Upper lower (n=367)	155 (42.2%)	114 (31.1%)	98 (26.7%)		
	Lower (n=33)	15 (45.5%)	18 (54.5%)	0 (0.0%)		

Association of awareness of diabetes and risk factors for diabetic retinopathy with socio-demographic variables according to age

As shown in Table 7, participants with diabetes mellitus who were younger than 55 years had a relatively high level of awareness (41.3%, n=126/305). Also, younger female participants had a significantly higher level of awareness (chi-square value: 33.6; p<0.01) than men, whereas in the 55+ age group, the opposite outcome was observed. The Christian community showed a higher level of awareness in both age groups. Participants from joint families showed a higher level of awareness in the age group below 55 years (chi-square value: 49.4; p<0.01). Elderly participants who were unmarried or those who had lost their spouse showed a significantly low level of awareness. Surprisingly, participants in both age groups from the upper class showed a relatively low level of awareness. Also, in the above 55-year age group, participants in the upper, lower, or lower socio-economic status groups had a low level of awareness (chi-square value: 59.2; p<0.01). It was observed that diabetes mellitus status, gender, religion, and socio-economic status were significantly associated with participants' awareness levels across both age groups (Table 7).

**Table 7:** Association of awareness of diabetes and risk factors for diabetic retinopathy with socio-demographic variables according to age-groups The data has been represented as frequency (n) and percentage (%); total sample N=854 for the age group <55 years and N=903 for the age group ≥ 55 years. SES: Socio-economic status; p < 0.05 was considered statistically significant for the chi-square test.

Variables	Categories	<55 years (n=854)					≥ 55 years (n=903)				
		n	Awareness level			Chi-square value (p-value)	n	Awareness level			Chi-square value (p-value
			Low (n=281)	Moderate (n=278)	High (n=295)			Low (n=399)	Moderate (n=273)	High (n=231)	
History of diabetes mellitus	No	594	214 (39.0%)	166 (30.2%)	169 (30.8%)	26.1(<0.01)	431	250 (58.0%)	89 (20.6%)	92 (21.3%)	66.5 (<0.01)
	Yes	305	67 (22.0%)	112 (36.7%)	126 (41.3%)		472	149 (31.6%)	184 (39.0%)	139 (29.4%)	
Gender	Female	342	90 (26.3%)	150 (43.9%)	102 (29.8%)	33.6 (<0.01)	406	201 (49.5%)	100 (24.6%)	105 (25.9%)	12.4 (0.002)
	Male	512	191 (37.3%)	128 (25.0%)	193 (37.7%)		497	198 (39.8%)	173 (34.8%)	126 (25.4%)	
Religion	Hindu	536	159 (29.7%)	215 (40.1%)	162 (30.2%)	89.3 (<0.01)	626	279 (44.6%)	216 (34.5%)	131 (20.9%)	132.0 (<0.01)
	Muslim	104	40 (38.5%)	28 (26.9%)	36 (34.6%)		157	73 (46.5%)	48 (30.6%)	36 (22.9%)	
	Christian	124	41 (33.1%)	4 (3.2%)	79 (63.7%)		56	5 (8.9%)	3 (5.4%)	48 (85.7%)	
	Others	90	41 (45.6%)	31 (34.4%)	18 (20.0%)		64	42 (65.6%)	6 (9.4%)	16 (25.0%)	
Place of residence	Rural	295	107 (36.3%)	95 (32.2%)	93 (31.5%)	2.7 (0.25)	351	163 (46.4%)	114 (32.5%)	74 (21.1%)	6.2 (0.046)
	Urban	559	174 (31.1%)	183 (32.7%)	202 (36.1%)		552	236 (42.8%)	159 (28.8%)	157 (28.4%)	
Type of family	Joint	255	69 (27.1%)	54 (21.2%)	132 (51.8%)	49.4 (<0.01)	338	151 (44.7%)	113 (33.4%)	74 (21.9%)	4.7 (0.09)
	Nuclear	599	212 (35.4%)	224 (37.4%)	163 (27.2%)		565	248 (43.9%)	160 (28.3%)	157 (27.8%)	
Marital status	Unmarried	168	30 (17.9%)	97 (57.7%)	41 (24.4%)	107.0 (<0.01)	121	62 (51.2%)	28 (23.1%)	31 (25.6%)	57.8 (<0.01)
	Married	580	210 (36.2%)	128 (22.1%)	242 (41.7%)		666	251 (37.7%)	225 (33.8%)	190 (28.5%)	
	Widow/ widower/separated	96	41 (38.7%)	53 (50.0%)	12 (11.3%)		116	86 (74.1%)	20 (17.2%)	10 (8.6%)	
SES	Upper	21	16 (76.2%)	3 (14.3%)	2 (9.5%)	76.5 (<0.01)	32	20 (62.5%)	12 (37.5%)	0 (0.0%)	59.2 (<0.01)
	Upper middle	195	46 (23.6%)	49 (25.1%)	100 (51.3%)		211	83 (39.3%)	63 (29.9%)	65 (30.8%)	
	Lower middle	494	196 (39.7%)	159 (32.2%)	139 (28.1%)		404	149 (36.9%)	133 (32.9%)	122 (30.2%)	
	Upper lower	144	23 (16.0%)	67 (46.5%)	54 (37.5%)		223	132 (59.2%)	47 (21.1%)	44 (19.7%)	
	Lower	-					33	15 (45.5%)	18 (54.5%)	0 (0.0%)	

Association of awareness of diabetes and risk factors for diabetic retinopathy with socio-demographic variables according to diabetes mellitus status

Among the non-diabetic group, those aged 55 years or older (chi-square value: 13.6; p=0.01) and men (chi-square value: 35.1; p<0.01) had significantly lower awareness of diabetes and its related factors. The Christian participants showed a significantly higher level of awareness, regardless of diabetic status (chi-square values: 142.7 for non-diabetics and 95.2 for people with diabetes; p<0.01). The non-diabetic participants from nuclear families (chi-square value: 19.1; p<0.01) and from rural backgrounds (chi-square value: 8.7; p=0.01) showed lower levels of awareness. Rural diabetics were significantly more aware than their urban counterparts (chi-square value: 24.0; p<0.01). In both groups, participants from upper socio-economic status showed a relatively low level of awareness. Also, participants in the upper, lower, or lower socio-economic status groups showed relatively low to moderate levels of awareness. It was observed that participants' awareness level was significantly associated with age, religion, place of residence, and marital status in both age groups (Table 8).

**Table 8:** Association of awareness of diabetes and risk factors for diabetic retinopathy with socio-demographic variables according to diabetes mellitus status The data have been presented as frequencies (n) and percentages (%); total sample sizes are N=980 for non-diabetics and N=777 for people with diabetes. SES: Socio-economic status; p < 0.05 was considered statistically significant for the chi-square test.

Variables	Categories	Non-diabetic (n=980)					Diabetic (n=777)				
		n	Awareness level			Chi-square value (p-value)	n	Awareness level			Chi-square value (p-value)
			Low (n=464)	Moderate (n=255)	High (n= 261)			Low (n=216)	Moderate (n=296)	High (n= 265)	
Gender	Female	453	196 (43.3)	143 (31.6%)	114 (25.2%)	13.6 (0.01)	295	95 (32.2%)	107 (36.3%)	93 (31.5%)	4.7 (0.097)
	Male	527	268 (50.9%)	112 (21.3%)	147 (27.9%)		482	121 (25.1%)	189 (39.2%)	172 (35.7%)	
Age	<55 years	549	214 (39.0%)	166 (30.2%)	169 (30.8%)	35.1 (<0.01)	305	67 (22.0%)	112 (36.7%)	126 (41.3%)	14.0 (0.001)
	>=55 years	431	250 (58.0%)	89 (20.6%)	92 (21.3%)		472	149 (31.6%)	184 (39.0%)	139 (29.4%)	
Religion	Hindu	653	292 (44.7%)	225 (34.5%)	136 (20.8%)	142.7 (<0.01)	509	146 (28.7%)	206 (40.5%)	157 (30.8%)	95.2 (<0.01)
	Muslim	115	73 (63.5%)	20 (17.4%)	22 (19.1%)		146	40 (27.4%)	56 (38.4%)	50 (34.2%)	
	Christian	122	44 (36.1%)	2 (1.6%)	76 (62.3%)		58	2 (3.4%)	5 (8.6%)	51 (87.9%)	
	Others	90	55 (61.1%)	8 (8.9%)	27 (30.0%)		64	28 (43.8%)	29 (45.3%)	7 (10.9%)	
Type of family	Joint	325	136 (41.8%)	74 (22.8%)	115 (35.4%)	19.1 (<0.01)	268	84 (31.3%)	93 (34.7%)	91 (34.0%)	3.1 (0.21)
	Nuclear	655	328 (50.1%)	181 (27.6%)	146 (22.3%)		509	132 (25.9%)	203 (39.9%)	174 (34.2%)	
Place of residence	Rural	418	219 (52.4%)	92 (22.0%)	107 (25.6%)	8.7 (0.01)	228	51 (22.4%)	117 (51.3%)	60 (26.3%)	24.0 (<0.01)
	Urban	562	245 (43.6%)	163 (29.0%)	154 (27.4%)		549	165 (30.1%)	179 (32.6%)	205 (37.3%)	
Marital status	Unmarried	147	49 (33.3%)	61 (41.5%)	37 (25.2%)	77.6 (<0.01)	142	43 (30.3%)	64 (45.1%)	35 (24.6%)	30.7 (<0.01)
	Married	688	307 (44.6%)	167 (24.3%)	214 (31.1%)		558	154 (27.6%)	186 (33.3%)	218 (39.1%)	
	Widowed/widower/separated	145	108 (74.5%)	27 (18.6%)	10 (6.9%)		77	19 (24.7%)	46 (59.7%)	12 (15.6%)	
SES	Upper	27	16 (59.3%)	11 (40.7%)	0 (0.0%)	121.2 (<0.01)	26	20 (76.9%)	4 (15.4%)	2 (7.7%)	119.0 (<0.01)
	Upper middle	231	73 (31.6%)	73 (31.6%)	85 (36.8%)		175	56 (32.0%)	39 (22.3%)	80 (45.7%)	
	Lower middle	503	238 (47.3%)	125 (24.9%)	140 (27.8%)		395	107 (27.1%)	167 (42.3%)	121 (30.6%)	
	Upper lower	201	137 (68.2%)	28 (13.9%)	36 (17.9%)		166	18 (10.8%)	86 (51.8%)	62 (37.3%)	
	Lower	18	0 (0.0%)	18 (100.0%)	0 (0.0%)		15	15 (100.0%)	0	0	

## Discussion

Over the past three decades, India's diabetes burden has significantly increased. Effective diabetes control and management require a combination of primary prevention through community-based health education, dietary counselling, and physical activity promotion initiatives, along with secondary prevention strategies such as systematic screening and prompt clinical management [12]. One of the most prevalent microvascular problems in diabetic patients is DR, which is also a major cause of visual impairment. According to population-based research, 10% of diabetes patients have vision-threatening DR episodes, while 33% of patients have indications of DR [14]. Lack of awareness on diabetic screening and the effect of diabetes on eye and retinal health often leads to poor health-seeking behaviour among people with diabetes, increasing DR prevalence and related complications [15]. The present hospital-based study included 1757 patients attending the eye OPD of a tertiary care centre in Northeast India to assess awareness of DM and its risk factors for DR, and to identify associated factors.

Most participants in this study were men and aged 54 years or older. The percentage of known diabetics was 44.2% (n=777), and half of the participants had a lower-middle-class socio-economic background. A moderate to high level of awareness of diabetes and risk factors for DR was observed in 1070 (61.3%) patients. The findings agree with other similar studies [16-18]. A study from South India reported that 55% of patients were unaware of specialised eye treatment for DR [19].

In the present study, known diabetics showed significantly more awareness of various risk factors and symptoms of DM. But both diabetics and non-diabetics were less aware of the lifestyle risk factors of diabetes. On the other hand, the age-group comparison revealed that the younger age group (below 55 years) was more aware of most risk factors and symptoms of DM. However, more than 75% of participants in both age groups were aware that DM can cause blurred vision. The difference in awareness might be attributed to the fact that complication-affected individuals with DM typically know more about the disease and its risk factors than people without complications [20]. Only 45% (n=350) of people with diabetes knew the typical range of PPBS levels, and both diabetics and non-diabetics had relatively little knowledge of the normal range for FBS levels and HbA1c. Low awareness of monitoring levels may affect patients' self-monitoring of blood glucose and periodic laboratory checks, leading to underdiagnosis or late diagnosis of the disease. Late diagnosis of type 2 diabetes may result in unchecked progression of DR and other micro- and macrovascular complications [21]. Suboptimal blood glucose management, hypertension, and dyslipidaemia are the traditional controllable variables linked to the development and progression of DR. The aberrant distribution of adipose tissue, as well as lifestyle factors such as diet, vitamin intake, exercise, and smoking, are among the less well-known modifiable factors that may be important [22]. The current findings suggest a lack of awareness among the participants regarding diabetes monitoring and prevention strategies, making them more vulnerable to DR and other major complications.

In the present study, awareness regarding DM and DR was significantly associated with age, DM status, religion, place of residence, family type, marital status, and socio-economic status. Previous studies have reported that older adults are more aware of DM, which contradicts our findings [23]. However, unawareness was more commonly reported in the non-diabetic group. In the present study, unawareness was more prevalent among the upper socio-economic class. A study from West Bengal reported that diabetes risks were much higher in the richest adults compared to the poorest adults due to a sedentary lifestyle, dietary habits and poor lifestyle behaviour. Policy-making to reduce economic inequality and awareness campaigns on diabetes care and treatment-seeking behaviour are important, particularly among older adults [24]. Similar to our findings, another study reported an association between Christianity and knowledge about DR. Higher educational levels among Christians were attributed to greater awareness among participants [25]. Awareness of DR was previously documented to be significantly correlated with gender, length of diabetes, and place of residence [17]. In the present study, urban diabetics showed a high level of awareness, while rural diabetics mostly showed a moderate level of awareness about diabetes and DR. Also, the awareness level was considerably low among the rural non-diabetics. The present findings support the observation of the Indian Council of Medical Research's India Diabetes Study [26]. Non-diabetics who were from joint families also showed a significantly high level of awareness. Many generations live together in joint families, which can make older family members more susceptible to chronic diseases like diabetes. One can gain more informal knowledge regarding diabetic complications, such as DR, by seeing the experiences and management practices of families with the disease.

The widowed, widowers, or separated individuals were observed to have lower awareness levels than their married counterparts, particularly those aged 54 or older (chi-square value=57.8; p<0.01) and non-diabetics (chi-square value=77.6; p<0.01). Widowed, widowed, or separated non-diabetic persons in India exhibit a notable lack of awareness of DM and DR due to their increased socio-economic vulnerability, restricted access to health information, and cultural barriers, especially for women [27]. In the current study, most participants were non-vegetarians. Previous studies have reported protective effects of fish and meat consumption on DR risk [28,29].

Limitation

The hospital-based study design limits the generalisability of the findings. The single-institution setting and the study's short duration limit its external validity. Moreover, the evidence in the current study is based on self-reported data. Future studies in multi-institutional settings or community-based longitudinal assessments could help gauge the level of awareness of diabetes and its risk factors for DR, as well as the factors that influence it, among the general population. Participants showed limited awareness of blood glucose monitoring levels, which might affect self-monitoring of blood glucose and periodic laboratory checks, leading to underdiagnosis or late diagnosis of the disease.

## Conclusions

A moderate to high level of awareness regarding diabetes and the risk factors for DR was demonstrated by the majority of participants. Individuals with a history of DM had a markedly higher level of awareness of the symptoms and risk factors of the disease. However, the lifestyle risk factors for diabetes were less well-known to both diabetics and non-diabetics. Patients' age, DM status, religion, place of residence, family type, marital status, and socio-economic position were found to be substantially correlated with their awareness of DM and DR. The majority of DM risk factors and symptoms were better known to the younger age group.

To reduce the prevalence of DR and enhance treatment planning, the results of this study could serve as a basis for awareness campaigns among the targeted patient group and medical professionals. To ensure that everyone in remote locations, especially those at risk, receives a dilated eye test at the time of first diagnosis and at least once a year thereafter, screening and awareness programmes are required.

## Appendices

**Table 9:** Questionnaire used for data collection

A. DEMOGRAPHIC PERFORMA						
Instruction: Kindly read the following statements carefully, select the best alternative from the given options, and tick (√) accordingly.						
Participant code number:						
Sl no.		Demographic details	Categories		Response	
1.		History of diabetes	(Yes/No)			
		If not, RBS				
2		Age (in years)				
3		Gender	Male			
			Female			
4		Religion	Hindu			
			Muslim			
			Christian			
			Others, please specify.			
5		Type of family	Nuclear family			
			Joint family			
6		Place of Residence	Rural			
			Urban			
7		Marital status	Unmarried			
			Married			
			Widow			
			Divorce			
8		Educational attainment of the household head (Modified Kuppuswamy scale, 2022)	Professional degree			
			Graduate			
			Intermediate or diploma (Class XI–XII)			
			High school (Class IX – X)			
			Middle school (Class V – VIII)			
			Primary school (Class I – IV)			
			Illiterate			
9		Monthly family income in rupees (Modified Kuppuswamy Scale, 2022)	> 185,895			
			92951-185894			
			69535-92950			
			46475 – 69534			
			27883-46474			
			9308-27882			
			< 9307			
10		Occupation of the head of the family	Legislators, senior officials & managers.			
			Professionals			
			Technicians & associate professionals			
			Clerical			
			Skilled workers & shop & market sale workers			
			Skilled agricultural & fishery workers			
			Craft & related trade workers			
			Plant & machine operators & assemblers			
			Elementary Occupation			
			Unemployed			
11		Dietary pattern	Vegetarian			
			Non-vegetarian			
B. Structured questionnaire for assessment of awareness of diabetes mellitus and risk factors for diabetic retinopathy. Instructions: Kindly read the following statements carefully and tick (√) the answer that is correct according to you. This information should be collected for all cases, including both DM and non-DM.						
Sl no.	Awareness statements			Response categories		Response
1	Diabetes mellitus is characterised by the following (please select one):			A group of diseases that result in low sugar in the blood		
				A group of diseases that result in too much sugar in the blood		
				A group of diseases that affect sugar in the blood		
				Not sure		
2	The risk factors for diabetes mellitus include			Family history (Yes/No)		
				Being overweight (Yes/No)		
				Physical inactivity (Yes/No)		
				Smoking (Yes/No)		
				Alcohol (Yes/No)		
				Intake of Junk Food (Yes/No)		
				Women on oral contraceptive pills (Yes/No)		
3	Symptoms of high blood sugar include			Frequent urination (Yes/No)		
				Increased thirst (Yes/No)		
				Extreme hunger (Yes/No)		
				Unexplained weight loss (Yes/No)		
				Blurred Vision (Yes/No)		
				Dry mouth (Yes/No)		
				Recurrent urine infection (Yes/No)		
4	Diabetes mellitus can be detected with the help of:			Sputum test (Yes/No)		
				Blood test (Yes/No)		
				Stool test (Yes/No)		
				Smear test (Yes/No)		
5	The food of a diabetic patient should include:			Daily products like milk and curd (Yes/No)		
				Vegetables like spinach, broccoli, salad, carrot, squash, and tomato (Yes/No)		
				Fruits like blackberries, oranges, apples, amla, guava, watermelon, and pomegranate (Yes/No)		
6	Patients with diabetes should steer clear of the following foods:			White bread, white rice and baked products (Yes/No)		
				Junk food (Yes/No)		
				Fruits that don’t contain sugar, like papaya, apple, star fruit, and mousumi (yes/no)		
				Brown bread and brown rice (Yes/No)		
				Fruits that contain natural sugar, like mango, banana, pineapple, and jackfruit (Yes/No)		
				Intake of alcohol (Yes/No)		
				Yoghurt with added sugar (Yes/No)		
7	Normal range for Fasting Blood Sugar (FBS) level is: (tick any 1)			90-100 mg/dL		
				70-110 mg/dl		
				70-90 mg/dL		
8	The normal range for Post Prandial Blood Sugar (PPBS) level is: (tick any 1)			< 140 mg/dl		
				140-200 mg/dL		
				> 200 mg/dl		
9	Normal range for % HbA1c level is: (tick any 1)			< 5.7 %		
				>5.7 - < 6.5%		
				> 6.5%		
10	There are ways to lower the risk of diabetes.			Dietary control (Yes/No)		
				Regular physical exercises (Yes/No)		
				Intake of alcohol & tobacco (Yes/No)		
				Regular health checkup (Yes/No)		
11	Obese people are more likely to develop diabetes. (Tick any 1)			True		
				False		
				Don’t know		
12	Prediabetes is considered a reversible condition (tick any 1).			True		
				False		
				Don’t know		
13	Diabetes can cause long-term damage in various parts of the body.			Kidneys (Yes/No)		
				Eyes (Yes/No)		
				Nerves (Yes/No)		
14	Diabetes patients should visit a physician at least (tick anyone):			Once a month		
				Once in 3 months		
				Once in a year		
				Twice in a year		
15	In diabetic patients, smoking causes (tick anyone):			Narrowing blood vessels		
				Widening of blood vessels		
				Injury to the blood vessels		
				Do not know		
16	Gestational diabetes occurs (if women) (tick anyone):			In pregnancy		
				At birth		
				After menopause		
				Before menopause		
				Do not know		
17	People with diabetes are prone to:			Pimples (Yes/No)		
				Chickenpox (Yes/No)		
				Urine infection (Yes/No)		
				Headache (Yes/No)		
18	Treatment of diabetes includes (tick any 1)			Insulin only		
				Oral Antidiabetic Drugs		
				Exercise		
				All of the above		
